# Circulating vitamin D and follicle-stimulating hormone levels are associated with subclinical atherosclerosis in climacteric women with menopausal syndrome

**DOI:** 10.3389/fendo.2026.1748598

**Published:** 2026-02-25

**Authors:** Xiaoting Yan, Lingyun Hui, Li Wang, E. Bai, Fen Li, Xuewen Yu

**Affiliations:** 1 Medical Department, First Affiliated Hospital of Xi’an Jiaotong University, Xi’an, Shaanxi, China; 2 Clinical Laboratory, First Affiliated Hospital of Xi’an Jiaotong University, Xi’an, Shaanxi, China; 3 Department of Obstetric and Gynecology, First Affiliated Hospital of Xi’an Jiaotong University, Xi’an, Shaanxi, China

**Keywords:** 25-dihydroxyvitamin D, climacteric women, follicle-stimulating hormone, intima-media thickness, menopausal symptoms

## Abstract

**Objective:**

Recent studies of climacteric women suggest that 25-OH vitamin D (25(OH)D) and follicle-stimulating hormone (FSH) levels may be associated with atherosclerosis. Whether interaction of 25(OH)D and FSH on subclinical atherosclerosis remains unknown. We investigated the relationship between 25(OH)D and FSH levels with carotid and right subclavian intima-media wall thickness (IMT), as well as it’s interaction on IMT, in climacteric women with menopausal syndrome.

**Methods:**

A total of 227 climacteric women aged 40~59 years from January 2023 to December 2024 were included in this study. Serum was assayed for 25(OH) vitamin D (25(OH)D), sex hormones, and traditional cardiovascular risk factors. The carotid and right subclavian IMT was evaluated using a B-mode ultrasound. Logistic analysis was used to estimate odds ratio (OR) and 95% CI for high IMT associated with 25(OH)D, FSH, and traditional factors. Andersson’s model was applied to evaluate the additive interaction between these factors.

**Results:**

The serum concentrations of 25(OH)D were inversely (OR: 0.945, 95 CI: 0.906-0.986) correlated with high IMT but FSH (OR: 1.021, 95 CI: 1.009-1.033) and fasting blood glucose (FBG) (OR: 2.302, 95 CI: 1.383-3.831) were positively associated with high IMT. After the stratified analysis by age, there were a significant multiplicative, but not additive, interaction between 25(OH)D and FBG on high IMT in women aged ≤50 years (p<0.05).

**Conclusions:**

High FSH and low 25(OH)D were associated with a high IMT except FBG. The formation of high IMT had different risk factors in younger and elder climacteric women with menopausal syndrome.

## 1 Introduction

Menopause is a major life event across a woman’s life span that can affect women in both short and long term. It included menopausal symptoms in the short term and an impact on bone and cardiovascular health over a long term. Of them, cardiovascular disease (CVD) is the leading cause of morbidity and mortality in women globally, and data from the American Heart Association have shown a notable increase in the risk for CVD after menopause (1). Previous researches found that early-onset menopause was associated with an increased risk of CVD and adverse cardiovascular outcomes in women (2). The prevalence of CVD was lower in premenopausal women compared with age-matched men, and it surpassed that of men during the perimenopausal and postmenopausal stages (3, 4). Changes of the vascular wall in women occurred at the very early stages of reproductive aging, which changes accelerated significantly after menopause. It was largely owing to the loss of protective effect of female sex hormones on the cardiovascular system (5, 6). Climacteric (40–60 years) women often exhibit a fluctuating decline in estradiol accompanied by an elevation in follicle-stimulating hormone (FSH). Prior studies assessed associations between FSH and subclinical CVD in women and results were inconsistent across these studies (7–11), yet the effects of endogenous sex hormones on cardiovascular health in climacteric women remain controversial (12, 13).

Vitamin D (VD) has a much wider range of biological functions. Data from several studies indicate that VD plays an important role in cardiovascular (CV) health, and low levels of 25-hydroxyvitamin D (25(OH)D) can negatively affect CV health (14). VD deficiency has contributed to various CV problems including hypertension (14), cardiac failure (15), myocardial infarction (16), coronary artery atherosclerosis (17), and peripheral arterial disease (18). However, there is the considerable controversy about VD attenuating cardiovascular disease in postmenopausal women. Studies suggested that healthy postmenopausal women with VD deficiency had increased risk of adverse cardiovascular outcome (19), and 25(OH)D was independently and negatively associated with carotid atherosclerosis in postmenopausal women with normal blood pressure and glucose tolerance (20). The results of other studies, however, show that there was no relation between VD and vascular health in postmenopausal women with metabolic syndrome (21), and little improvement in cardiovascular risk factors in postmenopausal women when supplemented with VD3 (22). Therefore, it is worth further exploring interactive effect of VD and endogenous sex hormone on cardiovascular health in climacteric women.

Noninvasive measurement of structural changes in carotid and subclavian intima-media thickness (IMT) is a well-established surrogate marker of early stages of CVD (23, 24). Increased IMT and presence of plaques are independently related to cardiovascular events (25). Thus, the aim of the present study was to analyze the association between carotid and subclavian IMT with serum concentrations of 25(OH)D and FSH in a sample of climacteric women who were diagnosed with menopausal syndrome.

## 2 Materials and methods

### 2.1 Study participants

**Figure 1:**
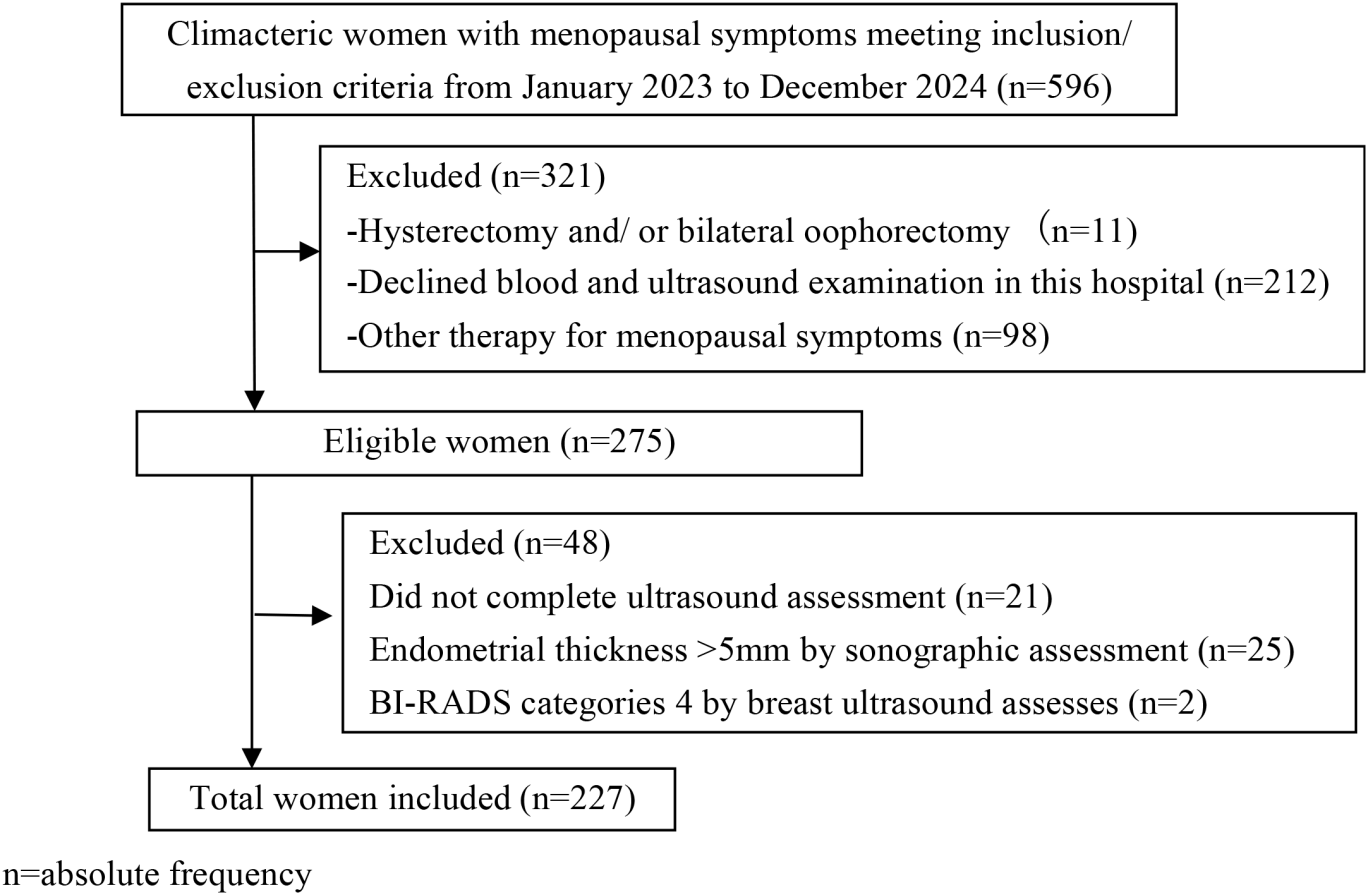
A flow chart for participant selection.

This cross-sectional study included 227 informed consenting women aged 40~59 years from the Menopause Clinic of the First Affiliated Hospital, Xi’an Jiaotong University between January 2023 and December 2024. The participant selection flowchart is shown in **Figure 1**. Before recruitment, women underwent the routine evaluation in our clinic including menopausal symptoms, serum sex hormones, blood liver-renal function, Lipid profiles, fasting glucose, blood coagulation tests, transvaginal sonography, and breast ultrasound. The inclusion criteria were (1) The perimenopausal and early postmenopausal stage (stages +1a, +1b, and +1c), which was determined by the Stages of Reproductive Ageing Workshop (STRAW) + 10 criteria (26). (2) experiencing menopausal symptoms, including vasomotor symptoms, sleep disturbance, mood fluctuations, and vaginal dryness. (3) not pregnant or breastfeeding, (4) no menopausal hormone therapy (HMT) use. The exclusion criteria included any of the following: (1) prior diagnosis of CAD, (2) history of thromboembolism, (3) history of diabetes mellitus or hypertension, (4) smokes, (5) alcohol abuse within the past year. All women gave informed consent for the use of their data.

### 2.2 Screening and physical measures

A detailed medical history was recorded for every woman who met initial eligibility criteria. Eligible women subsequently completed physical measures. Height and weight were measured. Body mass index (BMI) was calculated [weight (kg)/height^2^ (m)]. Systolic blood pressure (SBP) and diastolic blood pressure (DBP) were the average of 3 seated measurements taken using a YXY-61 medical electronic blood pressure monitor. The measurements of laboratory markers were obtained during their visit to a gynecologist.

### 2.3 Laboratory assay

Blood samples were obtained in the morning after at least a 12h overnight fast for the determination of levels of sex hormones including FSH, luteinizing hormone (LH), and estradiol (E2), total cholesterol (TC), triglycerides (TG), high density lipoprotein (HDL) cholesterol, low density lipoprotein (LDL) cholesterol, fasting blood glucose (FBG), 25(OH)D, homocysteine (Hcy), and D-dimer (DD). 25(OH) D levels were considered deficient at <20 ng/mL, insufficient at 20.0-29.9ng/mL, and sufficient at ≥30.0 ng/mL (27).

### 2.4 Ultrasound measures

A high-resolution B-mode ultrasound (Acuson Sequoia 512, America) equipped with a 8–14 MHz linear array transducer was used to measure carotid including bilateral common carotid artery, internal carotid artery, and right subclavian IMT and plaque. All arteries were scanned in both longitudinal and transversal sections. IMT was measured at a site approximately 1.0~1.5 cm proximal to the bifurcation of the carotid artery where it was free of discrete plaques. In the meantime, IMT was measured at the far wall of the proximal segment of the right subclavian artery. The mean IMT was calculated using two consecutive measurements on each artery. An IMT cutoff value of  ≥ 1.0 mm for carotid and right subclavian arteries or ≥1.2 mm for carotid bifurcation were considered intima-media thickening based on previous study (28). Plaques were defined as focal protrusion of wall into lumen with ≥1.5 mm IMT (28). All ultrasonographic measurements were performed by registered sonographer who holds a qualification certificate for large-scale medical instruments at the same time.

### 2.5 Statistical analysis

All statistical analyses were performed with SPSS 20 for Windows (Chicago, IL, USA). Quantitative variables were presented as means ± standard deviation ( ± SD) or median and min-max, depending on normality. The normal distribution of continuous variables was assessed using the Kolmogorov–Smirnov test. A student’s t test was used to compare each parameter of interest for independent groups if the data were normally distributed whereas the Mann-Whitney test was used for non-normally distributed parameters. Those variables that were found to be associated with IMT at p value ≤0.10 in the univariate analysis were put into the multiple logistic regression analysis. Firstly, binary logistic regression analysis was performed for evaluating the association between IMT (dependent variable) and independent variables, then subgroup analysis, further stratified by age (≤50 years, >50 years), were performed.

The multiplicative interaction between 25(OH)D and FBG, 25(OH)D and FSH, and FSH and FBG on IMT was assessed using logistic regression. To better understand the additive interaction between two variables on IMT, we dichotomized 25(OH)D, FSH and FBG at the threshold levels (defined as <20 ng/mL for 25(OH)D (low 25(OH)D), >40U/L for FSH (high FSH), and > mean of removing 5^th^ percentile for FBG = 4.8594 mmol/L (high FBG)) for analysis. To test for additive interaction, we followed the methods outlined by Andersson (29) to calculate the attributable proportion due to interaction (AP), the synergy index (SI), and the relative excess risk due to interaction (RERI) together with a 95% CI. If the CI of RERI and AP did not include 0 and CI of SI did not include 1, differences were considered significant, indicating an interaction between the two variables. All statistical tests were two-sided, and p-values <0.05 were considered statistically significant.

## 3 Results

In the cross-sectional study, the age of participants was 40–59 years old, with an average of 48.84 ± 4.51 years old. The proportion of normal IMT, thicker IMT, and plaque was 67.00% (152/227), 7.50% (17/227), and 25.60% (58/227), respectively. The proportion of high FSH (>40U/L) was 76.2% (173/227). The prevalence of vitamin D deficiency, insufficiency, and sufficiency was 54.20% (123/227), 33.00% (75/227), and 12.80% (29/227), respectively. The cohort was divided into two groups according to the IMT characteristics including normal IMT (n=152) and high IMT (including thicker IMT and plaque, n=75). Of them, there were 106 versus 33 (normal IMT vs high IMT) in women at age ≤50 years, and 46 versus 42 (normal IMT vs high IMT) in women at age >50 years.

### 3.1 CVD risk factors and carotid and/or right subclavian IMT in climacteric women

Characteristics of the study participants were shown in **Table 1**. Variables including age, BMI, TC, LDL cholesterol, FBG, 25(OH)D, FSH, and LH followed a normally distribution (P>0.05), while others including TG, HDL cholesterol, D-dimer, Hcy, estradiol, and FSH/LH radio showed a skewed distribution (P<0.05). Women with high IMT were relatively old and had a higher FSH, FBG, and FSH/LH ratio, while the 25(OH)D was significantly lower than women with normal IMT.

**Table 1:** Characteristics of the study participants according to the carotid and/ or right subclavian IMT.

Variables	Normal IMT (n=152)	high IMT (n=75)	*P* value
Age (years)^a^	48.09 ± 4.51	50.36 ± 4.15	<0.001^c^
BMI (kg/m^2^)^a^	22.53 ± 2.17	23.00 ± 2.78	0.200^c^
TC (mmol/L)^a^	5.09 ± 0.95	5.33 ± 0.89	0.068^c^
TG (mmol/L)^b^	1.05(0.76,1.60)	1.23(0.85,1.66)	0.212^d^
HDL cholesterol (mmol/L)^b^	1.45(1.30,1.68)	1.47(1.23,1.63)	0.832^d^
LDL cholesterol (mmol/L)^a^	3.06 ± 0.81	3.26 ± 0.79	0.068^c^
FBG (mmol/L)^a^	4.74 ± 0.59	5.13 ± 0.70	<0.001^c^
D-dimer (mg/L)^b^	0.47(0.40,0.56)	0.50(0.41,0.65)	0.061^d^
Hcy (umol/L)^b^	11.20(9.95,13.80)	12.00(9.05,17.80)	0.201^d^
25(OH)D (ng/mL)^a^	20.94 ± 8.14	17.60 ± 7.29	0.003^c^
Estradiol (pg/mL)^b^	18.40(8.25,46.55)	18.40(5.01,43.05)	0.394^d^
FSH (mU/mL)^a^	56.97 ± 28.69	75.22 ± 26.24	<0.001^c^
LH (mU/mL)^a^	37.91 ± 17.11	37.88 ± 11.82	0.991^c^
FSH/LH ratio^b^	1.64(1.27,2.04)	1.85(1.61,2.32)	<0.001^d^

a Data are expressed in mean values ± S.

b geometric means (interquartile range).

c Independent sample t-test.

d Mann Whitney test.

### 3.2 Multivariate analysis of carotid and/or right subclavian IMT in climacteric women

In multivariate logistic regression model the high IMT was associated with 25(OH)D, FBG, FSH, and age, whereas all other common cardiovascular risk factors missed significance (**Table 2**). Furthermore, there was no interactions between 25(OH)D and FSH on high IMT (data not shown).

**Table 2:** Multivariate analysis of presence of high IMT.

Variables	*B*	*S.E*	*Wals*	*P*	*OR(95% CI)*
25(OH)D	-0.0562	0.021	6.935	0.008	0.945 (0.906-0.986)
FBG	0.834	0.260	10.291	0.001	2.302 (1.383-3.831)
FSH	0.021	0.006	12.404	<0.001	1.021 (1.009-1.033)
Age	0.085	0.037	5.213	0.022	1.089(1.012-1.172)
constant	-9.292	2.215	19.124	<0.001	

### 3.3 Multivariate analysis of carotid and/or right subclavian IMT in climacteric women by age stratification

Since age may mask the effect of cardiovascular risk factors that increase with age, the multivariate regression was conducted separately by age (≤50 years, >50 years), which revealed a stronger association of 25(OH)D and FBG with high IMT for age ≤50 years, and FSH and FBG with high IMT for age >50 years. (**Table 3**).

**Table 3:** Multivariate analysis of presence of high IMT by age stratification.

Variables	*B*	*S.E*	*Wals*	*P*	*OR (95% CI)*
Age ≤50y					
25(OH)D	-0.070	0.027	6.568	0.010	0.932 (0.883-0.984)
FBG	1.018	0.403	6.375	0.012	2.767 (1.56-6.099)
constant	-4.732	1.900	6.206	0.013	
Age >50y					
FBG	0.953	0.369	6.681	0.010	2.593 (1.259-5.339)
FSH	0.037	0.010	12.785	<0.001	1.038 (1.017-1.059)
constant	-7.420	2.118	12.276	<0.001	

To explore the significant interaction on high IMT, the effects of 25(OH)D, FSH, and FBG were examined by the subgroup (age). There was a multiplicative interaction between 25(OH)D and FBG on high IMT in women at age ≤50 years (OR [95%CI]=1.040 [1.004-1.078], *P* for interaction = 0.030), and FSH and FBG in women at age >50 years (OR [95%CI]=1.008 [1.004 -1.012], *P* for interaction < 0.001). In addition, we tested for additive interaction between 25(OH)D and FBG, categorized as dichotomized at <20 ng/mL for 25(OH)D and >4.8594 mmol/L for FBG, on IMT in women at age ≤50 years. There was a 5.100-fold increased risk of high IMT for those with low 25(OH)D and high FBG, and a 3.500-fold increased risk for those with low 25(OH)D and low FBG compared to the referent group (**Table 4**). There was no evidence for an additive interaction between 25(OH)D and FBG (AP: OR [95%CI]=-0.012 [-0.240-0.214]; REPI: OR [95%CI] = -0.047 [-0.807-0.714]; SI: OR [95%CI]=0.983 [0.718-1.347]). The additive interaction between FSH and FBG on IMT was also analyzed in women at age >50 years. There was no significantly increased risk of high IMT for those with high FSH (>40U/L) and high FBG (>4.8594 mmol/L) compared to the referent group (FSH ≤ 40U/L and FBG ≤ 4.8594 mmol/L) (data not shown). Similarly, there was no evidence for additive interactions between FSH and FBG on high IMT in women at age >50 years (data not shown).

**Table 4:** Additive interactive analysis result.

Interaction variable		*n*	*B*	*S.E*	*Wald*	*P*	*O (95% CI)*
25(OH)D <20 ng/mL	FBG >4.8594 mmol/L						
–	–	38					1
–	+	29	0.796	0.700	1.295	0.255	2.217 (0.563-8.738
+	–	48	1.253	0.617	4.127	0.042	3.500(1.045-11.721)
+	–	24	1.629	0.676	5.806	0.016	5.100(1.355-19.192)

## 4 Discussion

The present work found a significant inverse association between 25(OH)D with high IMT, and positive association between FSH, FBG and age with high IMT in climacteric women with menopausal symptoms. Further stratified analysis by age, there were no significant associations between FSH with high IMT in women aged ≤50 years and 25(OH)D with high IMT in women aged >50 years. Although levels of VD, FSH, and FBG have all been hypothesized to relate to subclinical CVD that may represent unique vascular health risk factors in women, studies have not examined their joint impact upon IMT. Our study showed a significant multiplicative, but not additive, interaction between 25(OH)D and FBG on high IMT in women aged ≤50 years, and FSH and FBG in women aged >50 years. Although our study was the first to examine the joint influences of FSH, FBG, and VD upon IMT, several reports had examined associations between VD with IMT, as well as between FSH with IMT, as well as between FBG with IMT.

The result supported findings from other studies that low VD had been linked to CV health in many prior studies (14). One cohort study evaluating community-dwelling adults in the Northern Manhattan Study found 25(OH)D was inversely related to carotid IMT (β=−0.01 per 10ng/ml increase) (30). Other studies found 25(OH)D were independently associated with the incidence of carotid plaques in people with type 2 diabetes (31, 32). Alternatively, VD may have many direct beneficial effects on the vasculature which was supported by the expression of VD receptor in vascular smooth muscle cells and endothelial cells, and evidence from experimental studies that VD inhibited foam cell formation, suppressed vascular smooth muscle cell proliferation, and modified vascular endothelial function (33). Other experimental works showed in human umbilical vein endothelial cells that VD3 can induce a significant concentration-dependent increase in endothelial nitric oxide production (34), and in porcine aortic endothelial cells that VD3 can promote endothelial cells proliferation via nitric oxide dependent mechanisms (35). These findings indicated that VD played an important role in CV health.

Limited studies included women with different stages of the menopause transition assessed associations between FSH and subclinical CVD in women (7). Results were inconsistent across these studies with majority of those supporting an association between FSH and subclinical CVD. A large longitudinal multi-ethnic cohort study from SWAN showed that women (aged 42–52 years) experiencing either a medium or a high rise FSH before/after final menstrual period had significantly thicker carotid IMT than those who experienced a lower FSH rise after adjusting for socio-demographics and baseline CVD risk factors (8). A small sample study included 145 women aged 45–65 year observed a positive and statistically significant correlation between carotid IMT and FSH levels (r=0.21, P<0.009) (9). In contrast, a few of studies have reported a lack of association, or even an inverse relationship, between FSH and carotid IMT. A prospective population-based cohort study showed that higher FSH were related to lower mean carotid IMT among older postmenopausal women (64–73 years), independent of estradiol and BMI. However, the association between FSH with carotid IMT was not found among younger postmenopausal women (53–62 years) (12). Another a longitudinal study, each one log unit increase in FSH was associated with a 0.016 mm/year increase in carotid artery adventitial diameter progression (P = 0.003), independent of E2 level, but not associated with level or progression of carotid IMT among women at midlife (10). In a cross-sectional survey about relationships between the atherosclerotic plaque and the menopausal hormone levels among asymptomatic climacteric women did not show the association between FSH and carotid IMT (11). We observed a significant positive association between higher FSH with high IMT that was independent of E2 level in climacteric women with menopausal symptoms, especially for women over 50 years old. This finding suggested a strong effect of FSH on coronary vessels. Although we are not aware of how FSH was related to IMT, several studies reported that women experiencing menopausal vasomotor symptoms (VMS) are more likely to have adverse CVD risk profiles compared with women without VMS (4). A population-based prospective cohort from CARDIA reported an association between pattern of VMS with CVD events that women with a history of persistent VMS were more likely to have greater risk of CVD compared with women with minimal VMS (36). Another study to test trajectories of VMS in relation to subclinical CVD reports that women with early onset VMS had the highest migraine IMT (37). From our study and body of literature, it appears that FSH levels, particularly having menopausal symptoms, are associated with high IMT, although the exact nature of this relationship remains unclear. Of course, when considering whether FSH level increases a risk of developing high IMT, the issue of confounding factors between IMT and FSH level remains a concern.

In line with the presumption that carotid and subclavian IMT may increase due to the traditional cardiovascular risk factors, the study observed a significant positive correlation of FBG with high IMT regardless of age. Similar results were reported for the correlation of FBG with carotid IMT (38). In addition, one of the intriguing findings of this study was multiplicative interactions between 25(OH)D and FBG on high IMT in women aged ≤50 years. Low vitamin D level appeared to exacerbate the effects of high FBG on high IMT in women aged ≤50 years. In other words, the increased risk of high IMT seen with FBG was reduced when 25(OH)D was increased in younger women (≤50 years). When stratifying 25(OH)D and FBG into two categories, there was a 5.100-fold increased risk of high IMT for those with low 25(OH)D and high FBG compared to the referent group among women aged ≤50 years, however, there was no evidence for an additive interaction between 25(OH)D and FBG. This illustrated the importance of considering the VD and FBG interaction studies to keep vascular health among younger women. Recent studies show that the high circulating glucose can cause proinflammatory background in including interleukin-6 (IL-6) and tumor necrosis factor alpha (TNFα) in type 2 diabetes. The poorly controlled diabetic population expressed more increasing in inflammatory markers and carotid IMT than the well-controlled diabetic population. The increasing subclinical inflammation levels contribute to the development of vascular pathologies such as carotid atherosclerosis (39). On the other hand, VD also exhibits anti-inflammatory properties. It can suppress the production of proinflammatory cytokines including TNFα and IL-6. Therefore, VD deficiency can induce inflammatory response of the vessel wall which promote atherosclerosis (40). Furthermore, pancreatic β-cells express VD receptor (VDR), the binding of VD to VDR augments insulin synthesis and triggers insulin secretion thereby regulating blood glucose level. Additionally, the VD deficiency reportedly impairs islet insulin secretion (41). VD deficiency is very common in climacteric women and this undoubtedly exacerbates the onset of subclinical atherosclerotic process for this age group.

We also observed limited evidence of multiplicative interactions between FSH and FBG on high IMT in women aged >50 years. The magnitude of the association was small. This interaction should therefore not be over-interpreted. The relevance of FSH and FBG on high IMT remain to be clarified, further research focusing on this issue would be useful.

The present study has several limitations. First, one limitation was its cross-sectional nature, making it difficult to determine causation between low VD, high FSH levels and high IMT. Future research can design a prospective cohort study to follow up the study population for a long time, dynamically observe the changes of VD, FSH and the occurrence and development of IMT, and further confirm the relationship between low VD, high FSH levels and the change in IMT in climacteric women. Second, the analysis was confined to climacteric women with menopausal symptoms, while women with primarily quite healthy were excluded. However, this study cohort has been deliberately chosen as it represents the target population for which high IMT risk was important to individualize and initiated necessary measures to prevent high IMT in younger climacteric women. Thus, the findings and derived conclusions may not apply to relatively healthy women. Third, the sample size in our study was relatively small, thus, analysis of a larger population may have allowed to detect significant associations between VD, FSH and carotid and/or subclavian IMT and their differences with regard to age. Fourth, the climacteric women of this study were enrolled at the outpatient clinic of a single hospital within a period of time, which presents certain limitations. Carry out multi-center collaborative research, collect data from multiple hospitals to increase the representativeness of the population. Furthermore, in this context it can be kept in mind that there are more factors that may be worth considering as to high IMT risk as further observing indexes like postmenopausal years, age of menarche, sociodemographic determinants and lifestyle factors, which may add significant methods for preventing carotid/or and subclavian plaque risks.

## 5 Conclusions

Our findings suggested that low 25(OH)D, and high FSH, FBG levels and age are independently associated with high IMT in climacteric women with menopausal symptoms. The directions of these associations significantly differed by age. More importantly, a significant multiplicative interaction was observed between 25(OH)D and FBG on high IMT in women aged ≤50 years. The results suggest that formation of high IMT had different risk factors and pathogenic pathways in younger and elder climacteric women. Remarkably, this is a cross-sectional study. It is necessary to conduct further follow-up and researches.

## Data Availability

The original contributions presented in the study are included in the article/supplementary material. Further inquiries can be directed to the corresponding author.
